# A qualitative analysis of the perceived socio-cultural contexts and health concerns of sugar-sweetened beverages among adults studying or working at a post-secondary institution in Dharwad, India

**DOI:** 10.1186/s12889-021-11033-y

**Published:** 2021-05-29

**Authors:** Natalie Riediger, Anika Dhalla, Maureen Cooper, Andrea Bombak, Hemalatha Sreeramaiah

**Affiliations:** 1 Department of Food and Human Nutritional Sciences, Faculty of Agricultural and Food Sciences, University of Manitoba, Winnipeg, Canada; 2 Department of Community Health Sciences, Rady Faculty of Health Sciences, University of Manitoba, Winnipeg, Canada; 3 Department of Sociology, University of New Brunswick, Fredericton, NB Canada; 4 Food Science and Technology Department, University of Agricultural Sciences Dharwad, Dharwad, India

**Keywords:** Sugar-sweetened beverages, Diabetes, India, Health, Nutrition, Health policy, Global health, Stigma

## Abstract

**Background:**

We sought to explore the perceptions of the socio-cultural contexts and health concerns of consuming sugar-sweetened beverages (SSB) among Indian adults working or studying at a post-secondary institution in Karnataka, India.

**Methods:**

We completed a qualitative study, including 24 semi-structured interviews between 2017 and 2018 at the University of Agricultural Sciences, Dharwad, Karnataka, India. Data were analyzed using thematic content analysis.

**Results:**

One over-arching theme emerged, *westernization and changing perceptions of food, sugar, and health*. Participants discussed SSB and associated health concerns in the broad context of westernization and overall economic development in India. Three sub-themes regarding the health perceptions of consuming SSB were: *healthy drinks are clean and natural*; *hydration and energy;* and *moderation and body weight*. Hygienically-prepared beverages were a consistent concern among participants. Juices and beverages, such as tea or coffee, sweetened with jaggery were viewed positively due to their naturalness and lack of processed sugar. Participants perceived SSB as providing hydration and energy, particularly in hot weather. Lastly, if consumed in moderation, SSB were thought to have no direct adverse health consequences. Though some participants noted excessive, ‘addictive’ consumption would contribute to weight gain and diabetes.

**Conclusion:**

Perceived health concerns of SSB reflect dominant health issues in India, namely, food insecurity, food safety, and increasingly, diabetes. Policymakers tend to prioritize acute challenges over long-term concerns. As such, the capacity of any policy to address chronic nutritional concerns related to SSB are likely to be muted in the absence of improvements to food safety and security.

**Supplementary Information:**

The online version contains supplementary material available at 10.1186/s12889-021-11033-y.

## Background

Dietary sugar intake has rapidly become a global public health concern, with beverages identified as the major dietary contributor to sugar intake [1]. Organizations and researchers expressing health concerns frequently cite sugar-sweetened beverage (SSB) intake as being associated with weight gain and incident diabetes, though these studies have been mostly conducted with American populations [2, 3]. Furthermore, SSB intake is associated with dental caries, in a dose-response manner, as reported in a meta-analysis of studies from diverse populations [4]. The World Health Organization (WHO) has recommended policies, such as taxation, to address SSB intake in all countries in order to address a global increase in weight-related non-communicable diseases, such as Type 2 Diabetes [1]. A number of countries have implemented a SSB tax, including both high- and middle-income countries, such as, Mexico, South Africa, Philippines, Norway and United Kingdom [5]. India has not yet implemented any SSB tax to date, though consumption of sugar and SSB has been identified as a health concern in India, particularly among young people [6]; however, the importance of understanding local social, political, economic, and cultural contexts prior to considering SSB taxation as a viable intervention has been emphasized [7, 8]. Furthermore, biomedical models of disease, such as those for diabetes, developed within a western context may not align with individual perceptions or needs in low- or middle-income countries [9].

For the purposes of this paper, SSB refers mainly to soda or pop, energy drinks, fruit drinks, tea and coffee drinks, milk or milk alternatives, yogurt-based beverages, and any other beverage with any type of added sugar. This would exclude diet sodas, 100% fruit juices, plain or sparkling water, water flavored with non-caloric sweeteners, unsweetened coffee or tea, or any beverage without *added* sugar. While sweetened tea or coffee are not usually included in definitions of SSB, we have included them here because evidence suggests that these are highly consumed in India [10], though we are specific in this paper when discussing perceptions of certain beverages. Notably, juices, as well as other beverages, containing natural sugars, are often identified as health concerns given their natural sugar content [6, 11, 12], and as such, are also considered in our study. Despite the variety of sugary drinks, the types of beverages targeted by health policies or nutrition education programs have varied considerably across different jurisdictions and generally target only certain beverages, such as pop or soft drinks [13–15]. Importantly, food and drink carry important social and cultural meanings [16], which further underscores the importance of examining local meanings and understandings of the health concerns of consuming various SSB and naturally sweetened beverages. Qualitative inquiry focused on local understanding and experiences, is well-suited to examine how the development of global health policies, such as those addressing SSB, may shape, or be shaped, by local understandings of SSB.

There were an estimated 77.0 million adults with diabetes in India in 2019, placing it as the country with the second highest number of people with diabetes, behind China [17]. There are documented variations in diabetes prevalence among different geographical areas and socioeconomic groups, such that southern states and urban areas have higher prevalence [18]. Despite the high burden of diabetes, prevalence is projected to continue to rise, particularly among rural populations, and the increasing prevalence of diabetes has been characterized as a ‘time-bomb’ [19]. The relatively rapid increase in type 2 diabetes in India and the forecasted ‘time bomb’ is largely attributed to what is commonly referred to as ‘nutrition transition’ [20]. ‘Nutrition transition’ is characterized by increased population level intakes of fat (particularly animal fat) and sugar, which are accompanied by urbanization and economic development [21]. While weight status is a purported intermediary between nutrition transition and type 2 diabetes, a recent study of the Kerala Diabetes Prevention Program estimated that approximately one-third of the sample at high-risk of diabetes had a ‘normal’ body mass index (BMI) [22].

Though there is increasing concern over the public health implications of ‘nutrition transition’ and type 2 diabetes, India is often described as facing a double burden of both over- and under-nutrition [23]. Specifically, India continues to face persistent malnutrition, particularly for women and children [24], which is mainly attributed to extreme poverty and food insecurity. Furthermore, dental carries is also a major public health concern in India [10, 25], though research into the national prevalence remains scant [26]. However, unlike diabetes, prevalence of dental caries appears higher among lower socioeconomic populations and rural areas in India [10, 25], despite both conditions sharing SSB consumption as a risk factor.

Aside from health, India is one of the largest global producers of sugar cane [27] and as such, the crop is highly politicized [28]. It is well-known that food industry is often resistant to health policies aimed at SSB intake [29]. Furthermore, with sugar cane’s prominence in the local food system, sugar is found throughout a traditional Indian diet, in both food and beverages.

Given the increasing health concerns of SSB and their targets for policy and educational interventions in other jurisdictions, the potential for substitution effects [30], and the importance of social, economic, political, and cultural contexts to policy implementation and effectiveness, an in-depth exploration of local understandings of the health concerns of various SSB in India is warranted. India’s local food system, competing health demands and industrial interests make it a particularly pertinent site for such an exploration. As such, the purpose of this qualitative study is to explore the perceptions of the sociocultural contexts and health concerns of consuming SSB among Indian adults working or studying at an Agricultural post-secondary institution in Dharwad, a southern, rural Indian city.

## Methods

### Design

This qualitative study included face-to-face, semi-structured interviews conducted by four senior undergraduate nutrition students enrolled at the University of Manitoba, Winnipeg, Manitoba, Canada. Interviews were conducted over the course of two, three-month periods during the summers of 2017 and 2018 at the University of Agricultural Sciences Dharwad (UASD), Karnataka, India. This study was approved by the University of Manitoba Health Research Ethics Board (HS20625) and the UASD Institutional Research Ethics Committee, and carried out according to  their guidelines. All participants provided their individual informed consent prior to participating, though not all participants agreed to be quoted directly.

### Setting

Dharwad, Karnataka is an agricultural hub, as well as an academic one. Dharwad is also considered a twin city with Hubli, which in 2011 had a combined estimated population of 1.85 million [31]. Student researchers stayed at the UASD campus. UASD was established in 1986, with five distinct undergraduate programs, twenty-five Master’s programs, and eighteen Ph.D. programs in agriculture, ranging from business marketing, food technology and nutrition, to horticulture. The UASD student population includes approximately 4000 students and 450 faculty members [32]. Students are from all over India, though primarily from the home-state of Karnataka.

### Sample and recruitment

The inclusion criteria for interviews were that participants must be at least 18 years of age, an Indian citizen, and speak English. Participants were recruited through posters, which were posted across the UASD campus. Additional participants were recruited through snowball sampling.

### Data collection

Prior to being interviewed, participants completed a demographic questionnaire (Supplementary file 1), identifying their age group, sex, employment, and highest-level of education achieved. Interviews took place at a location of the participants’ choosing, usually around the UASD campus. Each interview was audio-recorded and transcribed verbatim. The semi-structured nature of the interviews allowed us to iteratively build on emerging themes by asking additional questions in subsequent interviews. Specifically, it became clear in early interviews that participants held different understandings as to what constituted sugary drinks. This required probing questions to examine how intake and perceptions might have varied between different beverages. The interview guide included questions pertaining to the beverages participants, family members, and community members consume; food patterns and availability in the local region; how patterns have changed over time; and their concerns and understandings of any health implications of various beverages (Supplementary file 2). The interview guide included additional questions pertaining to the taxation of sugary drinks; however, the research questions for the present study do not pertain to taxation.

### Analysis

Researchers repeatedly read the transcripts, developed a code list with the other student researcher in that year, hand coded the data, identified recurring meaning units, and clustered the meaning units into broader categories, and eventually themes. Student researchers also debriefed following each of the interviews, where in-depth discussion and reflection was used as a tool for thematic development. Study credibility was further achieved through regularly debriefing with the lead author (NR) and amongst the researcher-pairs themselves in each year regarding interview content, opportunities for further probing, and emerging themes [33].

Following the completion of data collection in 2018, two of the student researchers who collected the data in 2018 (second and third author) merged the two code lists from each year and re-coded and analyzed all data from both years with the organizational assistance of NVivo version 12. Each respective codebook was developed using a combination of pre-determined and emerging codes [34]. Following initial coding stages, axial coding techniques were used whereby codes were linked and interconnected into categories providing a basis for the emerging themes [35]. We further compared the results from the overall sample with the results reported in each year to further add to the trustworthiness of the study.

## Results

A total of 24 participants were interviewed, including 13 men and 11 women; fourteen interviews were conducted between June–July of 2017, and 10 interviews were conducted between June–July of 2018. Interviews ranged in length from 14 to 114 min, with a mean of 35 min (median 32 min). Briefly, the sample was relatively young with nearly half of participants between 18 and 25 years old and five participants were > 46 years old. The sample was also highly educated with all participants having some post-secondary education (*n* = 3) or a degree/diploma (*n* = 21), reflecting the study setting and inclusion criteria, as well as our recruitment strategy. Quotes are presented in the paper using pseudonyms.

The overarching theme of the analysis is *westernization and changing perceptions of food, sugar, and health*. Beverages, sugar, SSB, and associated health concerns were often discussed in the broad context of westernization, poverty, urbanization, as well as overall economic development in India. These issues influenced health perceptions of SSB by way of economic development in terms of purchasing power and who consumes SSB (or not), cultural values associated with food and beverages, and also the changing health issues in the country, which were attributed to westernization. While water was the most frequently consumed beverage and drinking water on campus was widely accessible, it was noted by several participants that access to drinking water was limited in nearby regions, which was related to economic development. Pop was viewed as a western dietary incursion whose intake has increased over time. Pop was perceived as being consumed more by affluent urban residents and younger people. Most participants mentioned younger people were more interested with 'western' culture in general, and SSB in particular. Three sub-themes emerged regarding the perceptions of the health implications of consuming SSB: *healthy drinks are clean and natural*; *SSB for hydration and energy;* and *moderation and body weight*.

### Healthy drinks are clean and natural

First and foremost, healthy beverages (and food) were perceived as clean and natural. The value of hygienically-prepared or clean food was a consistent point of discussion and concern among participants, not just specific to SSB, but in their diet overall.

*They should prepare the products [SSB] healthy way, hygienically then only all people will stay fit and healthy. –* Prabha

Furthermore, we noted food packages, including SSB, as well as food service establishments had specific labels or advertisements of their food being ‘hygienically prepared’. Two participants also expressed that cleanliness was a concern regarding soft drink processing, in that ‘urea’, ‘chemicals’, and other ‘impermissible substances’ may be present.

*If some companies add chemicals for those things, then it’s not good for human beings. –* Ganesh

In addition to cleanliness as a concern with respect to SSB, the ‘naturalness’ of the SSB or beverage was also a factor influencing perceptions. *Jaggery*, an unprocessed form of cane sugar, is often used to sweeten beverages like coffee or tea, and used in lesser quantities compared to the sugar content of soft drinks. Several participants also described *jaggery* as having important medicinal properties.

*So there are other drinks which don’t have sugars, and which are actually healthy. So there are a couple of drinks which the ingredient is actually jaggery, then the sugar. So the jaggery is actually going to be healthier and it has minerals and it’s not processed with dews of chemicals, as you know sugar is used – it contains a lot of chemicals due to processing. –* Rajesh

Some participants expressed distaste for processing, by emphasizing the importance of fresh ingredients or fruit-like ingredients. None of the participants identified juice as a health concern. Juices were viewed as a natural and healthy option for managing thirst and were considered comparable by Aditi to consumption of fresh fruit.

*Fresh fruit is not available all the time so for refreshing and getting instant energy they can consume the juices. No problem with that. But, like coming for carbonated beverages, like soda and everything, my opinion is drinking it every day is not good, for soda, carbonated beverages. No fruit content. That is not good. –* Aditi

The consumption of unnatural products was sometimes thought to be due to the limited availability of fresh options. One participant voiced concerns regarding the availability of consistent electricity and safety issues about storing fresh juice in a refrigerator, suggesting that while naturalness is important, cleanliness superseded naturalness. Finally, one participant expressed that perceptions of healthy food were changing with westernization, such that concerns regarding cleanliness and obtaining a sufficient amount of food were starting to be replaced with concerns about nutrition and the health implications of ‘eating out’.

### SSB for hydration and energy

Most participants identified SSB as providing ‘energy’, ‘hydration’, ‘carbohydrates’, and/or ‘electrolytes’. To be clear, these terms were mostly used positively and as meeting a nutritional need. With respect to hydration, pop, juice, and some other SSB were perceived as consumed more in regions that are hotter and/or during hot months, which was also reflected in pop being consistently referred to as a ‘cold drink’. Furthermore, sugar cane juice, made by roadside juicers, was also mentioned as an important source of hydration; the necessity of these stands, particularly when it is hot outside, were justified by providing people with needed hydration and electrolytes given the very hot weather and excessive perspiration.

*Whatever I told about my house, the same pattern is there also. And especially if we talk about drinks or cold drinks, they will be served … like, if someone is coming to meet you at your home, at that time they will do or if you are coming from so much of sunlight, because their weather is very much … like, temperate will be 45 to 55 degrees Celsius. So if you are living in a temperature with 45 to 55 degrees Celsius, definitely, you need to have some water. And at that time they used to prefer, especially in summers, cold drinks, cold drink, Maza, or Tang, these are the brand names which are being sold in India…..And then sugar cane. Sugar cane juice is more prominent in other part of India, because sweating will be more, loss of electrolyte will be more. So people used to consume lot of sugar cane juices.* - Garima

Malnutrition was identified as a health concern in India, related to poverty and limited economic development; as such, the importance of individuals acquiring sufficient energy was a strong undercurrent in the interviews. SSB were perceived as an energy source, though not providing considerable energy, but as Dhiraj explains, sugar overall is perceived as an important contributor to the Indian population’s caloric requirements, as well as its economy. In this case, self-sufficiency is related to India’s self-sufficiency in providing food security to its population.

*And part of self-sufficiency is supported by sugar; not only by rice and wheat and maize and stuff like this. If you take sugar out of our menu on a daily basis, I think half the Indian family – I'm not exaggerating; half the Indian family will not supplement their calorie requirement, fulfil their cal-… Because sugar, direct sugar consumption is supplying lot of calorie to their daily life, daily diet.* - Dhiraj

However, energy was not the only nutrient participants associated with SSB. One concern regarding nutritional inadequacy with SSB, particularly for children, was that pop may replace more nutrient-dense foods or would not provide other essential nutrients required for these populations. One participant also mentioned ‘bone problems’ from consuming too much pop, presumably because many SSB do not provide calcium. As previously mentioned, SSB were perceived to provide energy, but energy in the absence of other nutrients were not viewed as positively.

*“Most of the people say that it [pop] contains nothing, no nutrients, no carbs, no protein, no vitamins, no nothing, but contain some kind of carbs and it is having a flavour and it is also having – means it can put out the thirst. So I think it is not bad to have a certain drink because it is not putting on so much calorie. We can just give our good calories in our morning, so it is okay.”* - Gopal

While malnutrition and the need for energy was emphasized as an issue partially associated with poverty, Saumya explains that individuals living in poverty may consume SSB, partially for reasons of social status as well. Other participants also confirmed that pop had an aura of being ‘cool’, a ‘craze’, or being associated with higher social status. However, among those living in slums, Saumya perceived their consumption also reflected ignorance of their true nutritional needs, in addition to hunger.

*There are many people staying here in the slums and all and what I have noticed is not that they don’t have enough money now to eat, so the minimum things they can afford to eat but I realized they don’t have the knowledge about what to eat, what not to eat. And it’s like for them newfound elitism or whatever you want you can call it. So, they have a little more money in their hands and they feel like eating these packed chips and all those. So, there are some children in the slums living on that, you know? Not that they cannot afford good food, it is that they don’t have the knowledge. As long as their stomach is filled, they feel it is okay*. – Saumya

### Moderation and body weight

Participants largely spoke of pop and its health implications dismissively and in terms of moderation, both personally and in Dharwad specifically. Though pop was viewed as a western dietary incursion whose intake has increased over time, intake of SSB was still perceived to be low in Dharwad, specifically compared to both north India, more urban areas of south India, as well as higher income countries such as the U.S. and England. Because pop intake in Dharwad was perceived to be low, many participants did not view pop intake as a major health issue in Dharwad.

*I can't say exactly how much percentage of people are suffering with diabetes in India, but I don’t think carbonated sugar is contributing very highly to diabetes*-Gopal

Furthermore, pop was mostly perceived as something consumed socially and as associated with partying, particularly among younger generations, and mostly occurring in the evenings at hotels (i.e. restaurants) and social gatherings. Most participants did not report *regular* or daily consumption of pop. As such, many participants described their own intake as moderate, which they did not associate with adverse health effects unless intake was regular or daily.

*I think it is not good to consume daily. Sometimes when there is indigestion ... I mean, when we have heavy food ... but then the carbonated beverages is okay, like soda, that is okay but not every day.* -Aditi

*Whatever we eat it has some benefits and limitation like that I should say. If we drink too much also it may not be good if we drink Pepsi and all. Maybe have some health benefits if you drink too much but whatever we drink, we should drink in a normal level or appropriate level so that we should avoid this health reason. Something like that. But it’s not that we should not drink also. It’s also helpful in one way but we should not over-drink also. –*Aashima

When participants did identify health concerns of consuming too many SSB, weight gain and diabetes were mentioned, partially via their contribution to the frequently promulgated (if overly-simplistic) energy balance equation (‘obesity’ being produced by ‘calories in’ exceeding ‘calories out’) [36]. In this way, while increased energy intake was perceived as implicated in diabetes in India (‘calories in’) by some participants, so too were increases in sedentary lifestyles associated with stationary work (‘calories out’).

*Definitely it [SSB] is not at all good, okay, because it relates … it means … aggravates lots of issue and disorders, like, especially lifestyle disorders. So those who consume more I always use to advise not to consume more because it has some adverse effect, so one have to take care of it. And person to person it varies. But majority it has adverse effect on the body, especially weight gain, if you are not working that much, your consumption is more and work is less. So at that time one should avoid.*

*Interviewer: And can you give some examples of lifestyle disorders you mentioned earlier?*

*Respondent: Like, yes, diabetic people, especially pre-diabetic stages. There are so many people are obese, like, obese and other factors, like, to pre-diabetic stages, those are in pre-diabetic stage one should avoid cold drinks, especially carbonated drinks.*-Garima

Given the focus on the energy balance equation by several participants, there was discussion of diabetes and/or weight being a health issue an individual must and can control through diet, as illustrated by Garima’s quote. The importance of personal control was also expressed by participants reporting having diabetes or pre-diabetes. One participant expressed their feelings of guilt when describing how they try to control their food and beverage intake, and another expressed worry:

*We have a lot of time, so all this, drinking beer or soft drinks, sugary drinks comes only at night. And I know it’s not good for health because I eat a lot, drink a lot of sweet, come home and sleep. I worry about that. Next day, I drink two glass of warm water. I make mistake, I try to correct it.* – Dhiraj

Some participants also described sugar intake as an addiction, meaning a lack of control in quantity ingested, and felt this addiction contributed to adverse health via weight gain.

*Yeah, it has got an adverse effect on people’s health I think because the people, some practice it as a hobby to drink tea or these beverages but later they get addicted to it so much that while working they have to have tea for more than 10 cups. I think this adds amount of sugar in their body and it may lead to obesity gradually. So my opinion is to reduce as far as possible the consumption of these sugary drinks. -*Maddhav

Finally, it should be noted that some participants did not readily associate SSB with diabetes, which was thought to be more attributed to genetics or stress. Carbonated SSB were also mentioned by several participants as helping with digestion, particularly after heavy meals, which did not fit with any of the previously themes, but also did not merit its own theme.

## Discussion

SSB and their perceived health concerns were described by Indian adults working or studying at a post-secondary institution in Dharwad in the context of westernization. SSB intake was thought to have changed over time because of westernization, or rapid economic development, and that pop, specifically, was viewed as western. However, our results suggest that participants did not view SSB as a particularly salient health issue. The healthfulness of beverages, as well as food, was mainly attributed to their cleanliness and whether they were hygienically prepared. Health views of sugar varied according to level of processing, such that unprocessed sugar (ex. jaggery) was perceived as more healthful due to its medicinal qualities as compared to processed sugar used in pop. The perceived medicinal properties of products made from sugarcane are likely rooted in Ayurveda, the traditional Indian system of medicine [36]. SSB were also perceived as providing hydration, energy, and electrolytes. Consumption of SSB by children was potentially concerning due to its potential to displace more nutrient-dense foods rather than any inherent adverse health effect. This perception reflected the dominant nutritional concern, malnutrition. However, participants also linked SSB, if not consumed in moderation to increased weight and subsequently, diabetes. Health concerns of increased body weight were primarily viewed as a matter of personal responsibility and the importance of controlling one’s caloric intake and potential addiction. Interestingly, oral health was not mentioned by participants. This may reflect Miglani’s perspective that there is general lack of awareness of oral health as a serious public health issue in India [26].

The overarching theme of westernization as relating to changing patterns of dietary intake and health is aligned with academic literature pertaining to nutrition transitions [21]. While participants did not discuss food or beverage corporations negatively, with the exception of how it may relate to hygienically prepared foods or beverages, sales data for ultra-processed drink products demonstrate a central role of food industry in nutrition transition between 2002 and 2016 [37, 38]. It is likely that aggressive marketing practices of SSB in India may contribute to participant’s perceptions of pop or soda as ‘cool’ or a ‘craze’ [38, 39]. It should be noted, though, that only pop or soda were perceived as ‘western’ beverages by participants, which were not described as the most consumed SSB – sweetened tea was.

Participants’ concerns with food safety and cleanliness are similar to that reported by Surendran and colleagues [40] with respect to fruit and vegetables in peri-urban villages near Hyderabad, India, and among women living in Dehli with respect to food in general [41]. Gulati and colleagues [42] also report among mothers living in four Indian cities, that ‘healthy’ food was ‘hygienic’ food, which authors interpreted as a belief directing mothers to choosing more packaged, processed food that are subject to food safety standards [43]. Importantly, the public and policymakers generally prioritise acute concerns related to food safety over nutritional concerns or effects related to long-term intake [44, 45]. India is currently considering the implementation of front-of-package nutrition labels to identify packaged foods high in fat, sodium, and sugar [46], which may generate greater individual awareness of nutritional concerns.

There are myriad beverages available in Dharwad, including both SSB and non-SSB, with differential food safety concerns in different contexts. This is particularly relevant when considering substitution effects relating to efforts to reduce SSB intake. Indeed, substitution effects are acknowledged as a key consideration in SSB tax policy effectiveness [30], and are likely different depending on context. Importantly, Indian dietary guidelines would like individuals to “drink plenty of water and take beverages in moderation” [47]. While filtered water was readily available for respondents and on campus, the tap water was not safe for consumption, and notably, water insecurity was discussed by participants as prevalent among many neighbouring areas, and particularly in villages. Water security is a well-documented concern in rural India [48]. Programs or policies to reduce SSB intake in the absence of safe or hygienic alternatives are not likely to be effective. Furthermore, food insecurity is intertwined with water insecurity in that water is consumed for hydration, for preparing food hygienically, and for agricultural purposes [49]. Wojcicki and Heyman [50] discussed the importance of the Coca-Cola Company’s fortification of bottled water in sub-Saharan Africa. This fortification maintains the economic benefit the company provides to the region, whilst not contributing to added sugars in residents’ diet. However, the extent to which some populations pay for bottled water as compared to SSB may be limited and there is little evidence suggesting this may be effective.

Malnutrition was another health concern discussed in relation to food/beverages in the present study, which was attributed to poverty and food insecurity, and the potential for SSB to displace more nutrient dense foods. Again, similar to Walls’ research [44, 45], acute concerns take precedence over long-term or chronic nutritional concerns – although in our data, chronic nutritional concerns related to SSB were also present. In this regard, we argue that any policy approach to address concerns related to nutrition for chronic, non-communicable disease, including SSB, must grapple with food safety and water/food security issues, prior to, or in addition to nutrition security to maximize policy effectiveness, but also to minimize harm to those most vulnerable. One participant also linked sugar consumption in India to sugar production, highlighting how challenging it would be to reduce sugar consumption while also maintaining economic growth. This discussion echoes concerns related to the impact of tobacco control policies on tobacco farmers [51–53], including in Karnataka [53]. The links between sugar production and consumption in India has received less attention in the context of public health approaches to controlling sugar consumption. This gap merits further examination.

Participants also discussed increasing nutritional concerns related to weight gain and diabetes, with some participants’ discussions emphasizing personal responsibility in controlling weight or diabetes. Weight stigma may be attributable, at least in part, due to widely disseminated personal responsibility framing, such that higher-weight status is viewed erroneously as always unhealthy and a product of individual behaviors; as such, higher-weight individuals are often characterized as lazy, ignorant, or weak-willed [54, 55] . In turn, experienced and internalized weight stigma increases risk for stress and numerous physical and psychological health conditions [55]. Notably, commercial entities, including food industry, also promulgate a personal responsibility narrative [56]. A large, global study including 71 countries demonstrated significant and positive associations between higher national body mass index (BMI) scores and national wealth, independent of weight variables, on implicit weight bias [57]. These results suggest that country-specific weight stigma is indeed associated with economic development or westernization, suggesting that weight stigma in India may be projected to rise with increasing economic development. A recent Lancet Commission report on global health, nutrition, and climate change included a call for addressing weight stigma [58]. The Lancet Public Health further stated that, “policy makers should actively seek out the ways in which their policies could be stigmatizing” [59]. Therefore, the implications of policies directed at SSB on potential stigmatization must be explored.

While weight stigma was not a prominent finding in the current study, the utilization of personal responsibility framing and evocation of energy balance set the stage for weight stigma. Furthermore, despite participants largely acknowledging that higher income populations consumed more pop and that pop and its intake were influenced heavily by westernization, two participants suggested consumption of SSB among lower socioeconomic populations may reflect lower literacy levels or nutrition education. This may indicate how censorious social judgments regarding food intake and health can, and usually do, become directed at the most vulnerable. Importantly, in addition to weight stigma, hygiene and diabetes are also associated with pronounced social stigma in various global settings [60, 61]. These multiple stigmas related to food suggests the importance of cross-cutting frameworks to address stigma that transcend single conditions, as discussed by Birbeck and colleagues [62]. We join Kato and colleagues [63] in calling for more research of diabetes-related stigma informed by an intersectional lens and in different global contexts, particularly research examining stigma attributed to foods/beverages implicated in diabetes prevention and management, such as those high in sugar, including SSB.

### Limitations

This study is subject to limitations. The transferability of the findings to other populations in India are likely limited. The Indian population is highly heterogeneous regionally and the study sample was specific to an agricultural region and highly educated. The sample was further restricted to those who felt comfortable communicating in English, though notably, the university curriculum is taught in English. Regardless, results would have been enhanced had all participants been able to communicate in their first language. More research is needed to examine perspectives among those who regularly consume SSB, have lower levels of education and income, and who may have been diagnosed with type 2 diabetes.

## Conclusions

In conclusion, highly educated, English-speaking adults residing in a southern, rural Indian city perceived health concerns of SSB reflect the dominant food/health issues in the country, namely, food insecurity, food safety, and increasingly, diabetes. The capacity of any policy to address chronic nutritional concerns related to SSB are likely to be muted in the absence of improvements to food safety and population food/water security. Global economic forces were acknowledged by participants as contributing to changing diets, and specifically SSB intake, in India. Some participants described food, weight, and diabetes as a matter of personal responsibility. In this regard, SSB policies may inadvertently contribute to the growing problem of global weight stigma, and its adverse associated health outcomes. Notably, structural improvements in food and water security would improve population health and are not targeted at individuals nor would they utilize moralizing discourses. However, more research is needed to explore weight and diabetes stigma among different socioeconomic groups in India as well as its potential intersections with sugar intake and SSB.

## Supplementary Information

**Additional file 1.** Participant demographic information

**Additional file 2.** Interview Guide

## Data Availability

The data that support the findings of this study are available from the corresponding author but restrictions apply to the availability of these data, which were used under license for the current study, and so are not publicly available. Data are however available from the authors upon reasonable request and with permission of the University of Agricultural Sciences, Dharwad.

## Abbreviations

SSB: Sugar Sweetened Beverage
WHO: World Health Organization
BMI: Body Mass Index
UASD: University of Agricultural Sciences Dharwad
