# Supplementary material for: A qualitative analysis of the perceived socio-cultural contexts and health concerns of sugar-sweetened beverages among adults studying or working at a post-secondary institution in Dharwad, India
Source: BMC Public Health. 2021 May 29;21:1016. doi: 10.1186/s12889-021-11033-y (PMC8164752; doi:10.1186/s12889-021-11033-y)
Supplement: Supplementary file 1 — Additional file 1. Participant demographic information [file 12889_2021_11033_MOESM1_ESM.docx]

**Participant demographic information**

Date:________________________

Participant ID number: _________

1. Age
   1. 18-25 years old
   2. 26-35 years old
   3. 36-45 years old
   4. ≥46 years old
2. Sex
   1. Male
   2. Female
   3. Other
3. Please select your employment status
   1. Employed full or part-time for wages
   2. Unemployed and looking for work
   3. Unemployed and NOT looking for work
   4. Student
4. What is your highest level of education?
   1. Less than high school graduation
   2. High school graduation
   3. Some post-secondary education
   4. Post-secondary graduate (degree or diploma)
5. How would you rate your health compared to others your age?
   1. Excellent
   2. Very good
   3. Good
   4. Fair
   5. Poor
6. How often do you consume sugary drinks (such as soda/pop (regular or diet), sports drinks, sweetened tea, sweetened yogurt drinks)
   1. Never
   2. Less than once per week
   3. 1-3 times per week
   4. 4-6 times per week
   5. At least once per day
