# Supplementary material for: A qualitative analysis of the perceived socio-cultural contexts and health concerns of sugar-sweetened beverages among adults studying or working at a post-secondary institution in Dharwad, India
Source: BMC Public Health. 2021 May 29;21:1016. doi: 10.1186/s12889-021-11033-y (PMC8164752; doi:10.1186/s12889-021-11033-y)
Supplement: Supplementary file 2 — Additional file 2. Interview Guide [file 12889_2021_11033_MOESM2_ESM.docx]

**Date:______________**

**Participant ID:____________________**

**Interviewer:______________________**

**Interview Guide:**

1. Student researcher to state participant ID number.
2. Tell me a little about yourself. Who are you and what do you do?
3. What is your life like outside of work/school?
4. Is your life like others living in Dharwad?
5. What do you normally eat at home? Work/school?
6. What do other people in Dharwad eat?
7. Do you drink the tap water?
   1. Why or why not?
   2. How does that make you feel?
   3. How do you cope with that?
   4. What do you drink instead? Why?
8. Where do you get your food?
   1. What challenges do you encounter, if any, in acquiring the food you want?
9. Where do others in Dharwad get their food?
10. Do people have trouble getting food?
11. How do you feel about your diet? Are you able to eat what you like?
12. What about the diet of other people in Dharwad?
    1. Prompt: do you consider the people of Dharwad healthy?
13. Do you ever drink sugary drinks? Why or why not?
14. Are sugary drinks commonly consumed in Dharwad?
    1. How do you feel about that?
15. What have you heard about a proposed tax on sugary drinks?

If not, prompt: the WHO has recommended that countries adopt a tax on sugary drinks.

1. Why do you think the WHO has made this recommendation?
2. How do you feel about that?
   1. Prompt: do you agree or disagree with a tax? What information would you need to know to make a decision?
3. Who do you think the tax is targeting?
4. Who might be impacted by this tax?
   1. Poor or rich?
   2. Obese?
   3. Young or old?
5. What, if any, special considerations should be made for India as compared to other countries if a tax is implemented?
6. A tax of this type would generate a lot of revenue. Where do you think revenue WOULD go?
   1. How does that make you feel?
7. What do you wish would be done with revenue if a tax is implemented?
8. Are you supportive of this type of tax? Why or why not?
9. Is there anything else you think I should know?
